# Oxidative, biochemical and histopathological alterations in fishes from pesticide contaminated river Ganga, India

**DOI:** 10.1038/s41598-022-07506-8

**Published:** 2022-03-07

**Authors:** Zeshan Umar Shah, Saltanat Parveen

**Affiliations:** Limnology Research Laboratory, Department of Zoology, Aligarh Muslim University, Aligarh, 202002 India

**Keywords:** Environmental sciences, Natural hazards, Biomarkers, Risk factors

## Abstract

The river Ganga basin accommodates large scale of agricultural activities, where large quantities of pesticides are applied. To assess the biological impact of pesticide residues that are continuously entering in the water body, biomarkers are common approach in bio-monitoring study as early warning signals to pollutants. In the present study qualitative and quantitative analyses of gill and liver histopathological and the biochemical parameters were determined in *Rita rita* and *Cyprinus carpio*. The difference in the histopathology and oxidative stress responses emphasize the response of selected fishes to the presence of contaminants in the water. Sloughing of lamellar epithelium and their disruption, dilated vessels, lamellar fusion, smooth muscle hypertrophy in the gill and necrosis, Vacuolation in hepatocytes, inflammation and distorted arterial walls were seen in the liver. The biochemical parameters were the main contributors to discriminate the changes in the fish physiology. In conclusion, the gill and liver histopathological responses, although not reflecting specific contaminants, but can be used as biomarkers of environmental contamination.

## Introduction

Agrochemicals are used in the fields for pest control, about 90% of these chemicals are left out in the environment without degradation. The bio-geographical structure of river Ganga has changed by large scale of anthropogenic activities such as usage of large quantity of pesticides in the agricultural areas along the basin^1–3^. Based on earlier findings^3–7^ these chemicals persist in the compartments for years causing impact on non-target organisms^3,7^ under altered physico-chemical parameters^4^ thus the structure and functioning of the cell may change. These changes will influence the well being of animals at population and ecosystem^8–10^. Biomonitoring in order to assess ecosystem integrity at regular basis is essential for management of aquatic ecosystem. The biomarkers such as oxidative stress markers although non-specific has proved to be meaning indicators of health of both marine and fresh water for it is responsible for the alteration in bio-molecules. The damage to proteins or different class chemical modifications of amino acids during oxidative stress can give rise to protein carbonyls^11^. It has been suggested that protein carbonyl induction may serve as surrogate biomarker for general oxidative stress^12^. Although various reports of oxidative stress in fish model in response to pollutants^13–15^ have suggested various enzymatic and non-enzymatic antioxidants as biomarkers of oxidative stress. Nevertheless, very less number of studies has been reported on protein carbonyl in case of fish.

Histological changes in the organism exposed to the contaminants have been considered as the best tool for evaluating the toxic effects both in laboratory and field studies^16–19^. Gills as primary organs for oxygen uptake in fish which remain in continuous contact of toxicants present in water, thus the stress is exacerbated. Gill lesions as indicative of toxicant effect have been previously used in various laboratory and field studies^20–23^. Liver the organ primarily meant for detoxifying, glycogen storage and release of glucose to the blood, synthesis of several components of blood plasma, shows histopathological changes in fish upon exposure to contaminants^24–26^.

Thus in the backdrop of above cited literature, histopathological changes in gills and liver upon toxicant exposure are useful tool to assess the impact of the toxicity in vital processes of a living organism. The present study was carried out to evaluate oxidative stress, biochemical changes and histopathological changes in the two commonly edible fishes collected from two different sites of pesticide contaminated river Ganga.

## Material and methods

### Chemicals and reagents

Bovine serum albumin (BSA), 2,4-dinitrophenyl hydrazine (DNPH), guanidine hydrochloride and TCA used were purchased from Sigma Aldrich. Other chemicals and reagents of high quality and purity were purchased locally.

### Collection of samples

**Figure 1:**
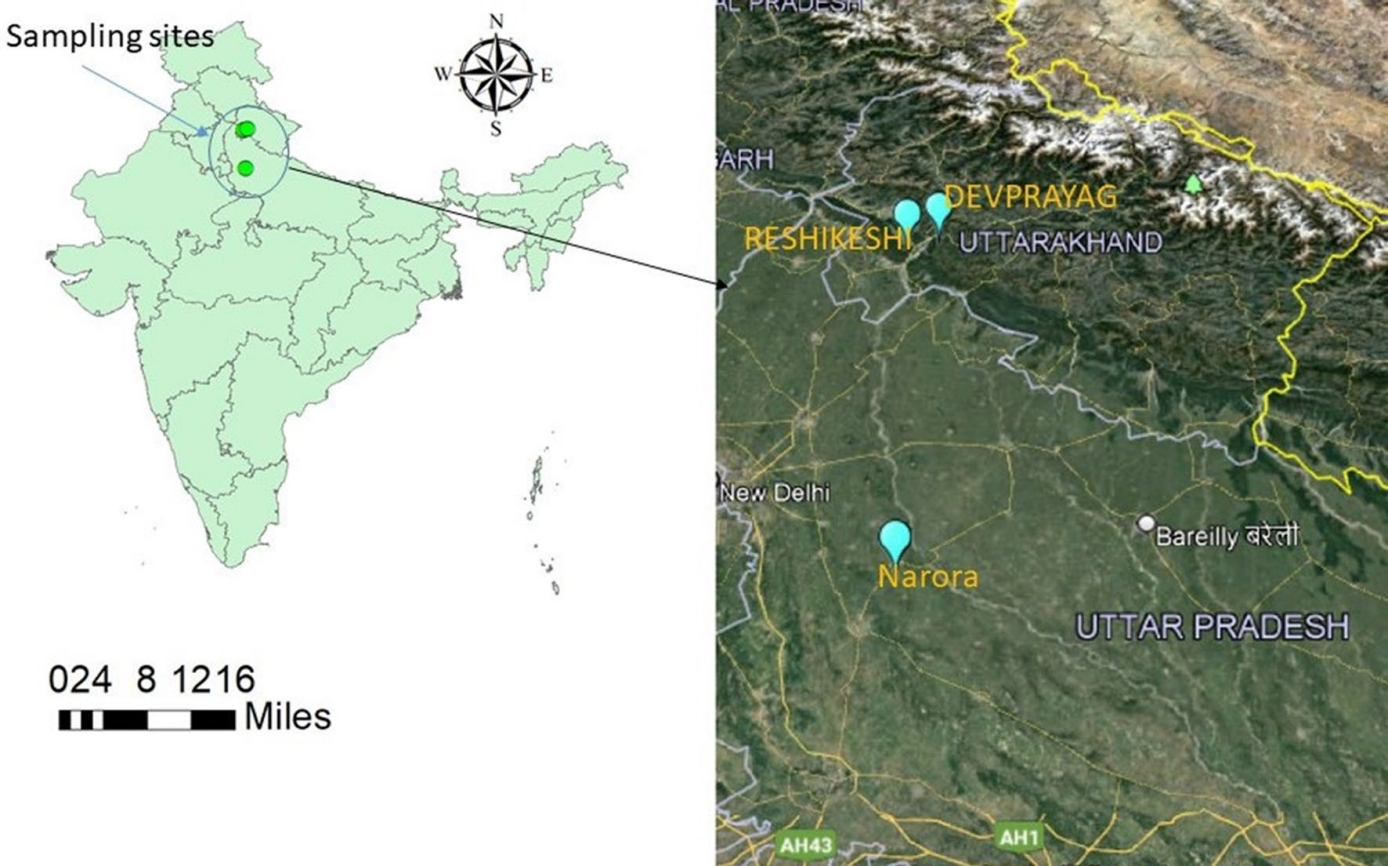
Map showing sampling stations (Devprayag, Rishikesh and Narora) along the length of river Ganga (source Google earth).

The investigation of the fishes *Rita rita* and *Cyprinus carpio* procured from river Ganga at middle station (Narora, 28^o^12ʹ02ʹʹN,78^o^23ʹ41ʺE), upper station (Rishikesh, 30^o^03ʹ51ʹʹN,78^o^17ʹ28ʺE) and Devprayag (30^o^08ʹ54ʹʹN,78^o^35ʹ35ʺE) as control group with the help of local fisherman in August 2019 (Fig. 1). The twenty five fishes of each species at each site were collected and only 10 were selected for the study. The biometric data of the fishes is mentioned earlier in^7^. Physico-chemical properties of the water samples were analysed following the procedures of APHA^27^ (Table 1). After collection, fishes were decapitated and transferred to the ice box. The packed box was immediately brought to the laboratory and followed by dissection tissue samples were obtained.

**Table 1:** Physico-chemical parameters of water samples collected from Narora and Rishikesh sites of river Ganga.

Parameters	Middle stretch (Narora)	Upper stretch (Rishikesh)	Bureau of Indian standards (BIS and ICMR) acceptable limit
Temperature (℃)	23.3 ± 1.52	20.5 ± 0.75	NA
Dissolved oxygen (mg/l)	2.03 ± 0.15	3.24 ± 0.66	5
Carbon dioxide (mg/l)	9.5 ± 1.21	4.4 ± 1.09	NA
pH	7.3 ± 0.02	8.2 ± 0.13	6.5 – 8.5
Biological oxygen demand (mg/l)	18.4 ± 1.65	12.1 ± 0.93	5
Chemical oxygen demand (mg/l)	26.8 ± 2.46	18.3 ± 1.19	NA
Conductivity (µS cm^-1^)	386 ± 17.9	190 ± 19.6	300
Nitrate (mg/l)	0.07 ± 0.01	0.02 ± 0.01	45
Phosphate (mg/l)	0.08 ± 0.03	0.05 ± 0.01	NA
Total dissolved solids (mg/l)	415 ± 0.81	281 ± 0.75	500

± Standard deviation.

NA = Not available.

### Oxidative stress assay

For estimation of stress biomarkers gill and liver tissues of selected fishes were removed, washed with phosphate buffer and 10% homogenate was prepared using 0.1 M phosphate buffer with pH 7.4 in a Teflon homogenizer. Lysed homogenate was centrifuged at 10,000 rpm for 20 min at 4 ℃. After centrifugation both pellet and supernatant was obtained and kept at -20 ℃ until analysis.

### Catalase (CAT)

Enzyme CAT was measured as per the protocol of Claiborne^28^. Dismutation rate of H_2_O_2_ to water and molecular oxygen is proportional to the concentration of catalase.

### Superoxide dismutase (SOD)

SOD activity was measured using the protocol of Marklund and Marklund^29^. The method is based on the inhibition of auto-oxidation of pyrogallol by SOD activity.

### Glutathione-S-transferase (GST)

Activity of GST was measured by adopting the protocol of Habig^30^. In this method reaction between 1-chloro 2, 4-dinitrobenzene (CDNB) and glutathione (GSH) results in the synthesis of CDNB-GSH conjugate.

### Protein carbonyl assay

Protein carbonyl was analysed following the procedure of Levin^31^ as modified by Floor and Wetzel^32^. Soluble protein (0.5 ml) was reacted with 10 mM DNPH in 2 M hydrochloric acid for 1 h at room temperature and precipitated with 6% trichloroacetic acid (TCA). The obtained pelleted protein was washed three times in ethanol/ethyl acetate (1:1) solution. Protein was then solubilised in 6 M guanidine hydrochloride solution, 50% formic acid and centrifuged for 5 min at 16,000 × g to remove any trace of material. The protein carbonyl content was measured spectrophotometrically at 366 nm.

### Protein estimation

Protein estimation was done by Lowry^33^ method using Folin-Ciocalteu regent and BSA as standard.

### Histolopathology

Tissue processing, block formation and staining were done following Gray’s^34^ method. Histological examination was carried out to access the changes in the tissues. Tissues were collected and fixed in the 10% formalin solution. Post fixation the tissue was washed several times with distilled water to remove any excess of fixative and subsequently dehydrated in different ascending grades of alcohol. Cleared in xylene, the dehydrated samples were transferred to xylene wax, followed by imbedding in pure molten wax. Wax blocks of samples, prepared in cavity blocks pre-coated with glycerine were obtained in warm water. Five micron thick sections of the sample tissues were obtained using rotary microtome (Leica RM 2125 RTS) and affixed to alcohol washed clean glass slides pre-coated with Mayor’s albumin and allowed to dry at room temperature for 12–16 h. The section was deparaffinised followed staining with Harris haematoxylin–eosin (HE) for histological studies.

### Statistical analysis

The data were analysed statistically by analysis of variance (ANOVA) followed by Duncan’s Multiple Range Test (DMRT). The significance was accredited at P ≤ 0.05 and all the results are presented as mean ± standard error of the mean.

## Results and discussion

The physicochemical conditions of the selected stations were evaluated by analysing ten parameters. The mean value for each station of river Ganga is given in (Table 1) and compared with national standards (BIS and ICMR). Dissolved oxygen was below the set value of 5 mg/l (ICMR) at both the stations suggesting the water is polluted with contaminants. BOD in general gives the qualitative index of the organic substance which is degraded quickly in short period of time. High BOD values at both the stations are possibly due to large proportion of agricultural, domestic sewage and organic load. Low pH at Narora station may be attributed to high degree of organic load by bacteria at high temperature resulting in the increased level of carbon dioxide in water which in turn leads to low pH concentration.

**Figure 2:**
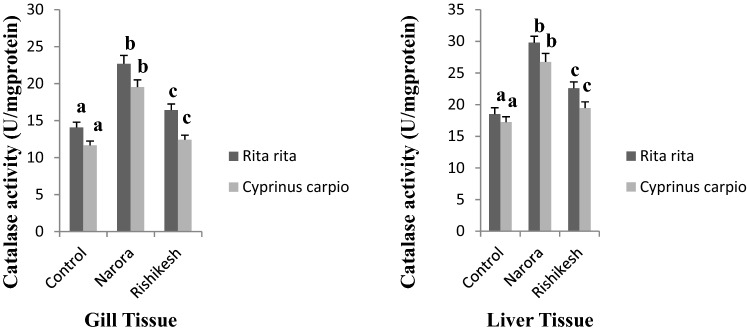
Catalase activity in gill and liver tissues of *Rita rita* and *Cyprinus carpio* from Narora and Rishikesh. The values are expressed as mean ± SE (n = 10). Values are expressed as U/mg of protein. The significance levels observed is P ≤ 0.05 when compared with the control group.

Difference in biochemical analyses shows the evidence of oxidative damage. Two different sites were compared for oxidative damage due to the pollution of pesticides. A significant increase in the catalase activity was observed in gill and liver of both *R. rita* and *C. carpio* from both the studied stations (Fig. 2) compared to control fishes capture from Devprayag station. Fishes captured from Narora station was found to have higher CAT activity than fishes captured from Rishikesh station due to higher pesticide pollution. High CAT activity results in high production of H_2_O_2_. Similar results have been reported by many workers^35^ reported high CAT activity in *Gambusia affinis* after lethal effect of an organophosphate pesticide, monocrotophos. Khare^36^ has observed significant increase in *Catla. catla* after pesticide exposure. Clasen^37^ has reported increase in liver catalase activity of *C. carpio* from pesticide contaminated rice fish system. Increase in the CAT activity reflects oxidative damage, responses to contaminants and mechanism of repair by which the organism protects themselves by toxicity of chemicals.

**Figure 3:**
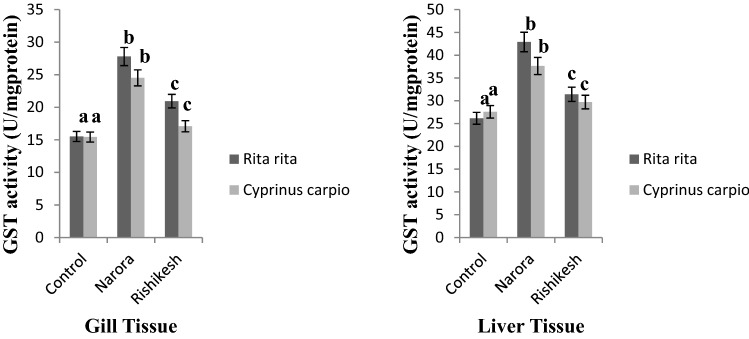
GST activity in gill and liver tissues of *Rita rita* and *Cyprinus carpio* from Narora and Rishikesh. The values are expressed as mean ± SE (n = 10). Values are expressed as U/mg of protein. The significance levels observed is P ≤ 0.05 when compared with the control group.

The GST activity was found high in both liver and gill tissues of *R. rita* and *C. carpio* at both the studied stations (Fig. 3) compared to control group. The GST activity was found significantly higher at Narora station in both the fishes compared to fishes captured from Rishikesh station. Maximum GST activity in different tissues of fishes has been reported by many workers, Clasen^37^ has reported high GST activity in Liver, gill and brain of *C. carpio* from pesticide contaminated rice fish system. The induction of antioxidant enzymes, such as CAT and GST, can be seen as a significant adaptation to pesticide induced pollution stress^38^. These enzymes have a high capability for scavenging pesticide compounds by converting them into an easily excretable form. Some earlier studies also show significant increase in the GST activity of the fish exposed to the pesticides and other chemical pollutants^39,40^.

**Figure 4:**
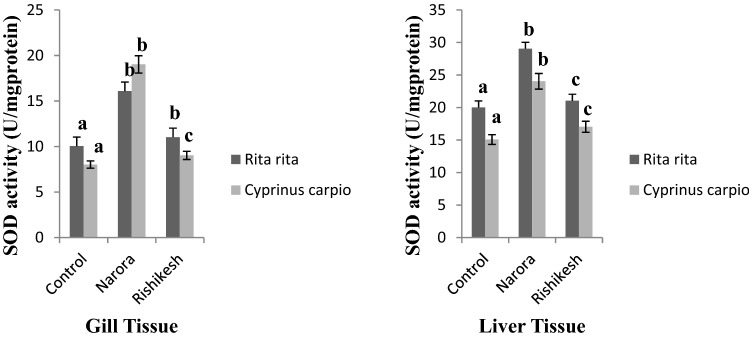
SOD activity in gill and liver tissues of *Rita rita* and *Cyprinus carpio* from Narora and Rishikesh. The values are expressed as mean ± SE (n = 10). Values are expressed as U/mg of protein. The significance levels observed is P ≤ 0.05 when compared with the control group.

SOD is a group of metalloenzymes that act as antioxidants and are the principal defensive mechanism against the damaging effects of superoxide radicals in living organisms^41^. In this study the SOD activity (Fig. 4) in the liver of the two fishes procured from Narora and Rishikesh was found significantly higher than two fishes captured from Devprayag station of the river Ganga. The SOD activity in the gill tissue of the fishes from Narora was found higher than the fishes captured from Rishikesh station of the river Ganga. Due to the presence of enormous quantity of pesticide pollution at Narora station increased SOD activity provides first line of defense against oxygen radicals, the elevated level of antioxidant enzyme demonstrates pesticide pollution induced adaptation in the fish and is an attempt to neutralize the generated ROS molecules. Similar results have been found by many workers, Kavitha and Rao^35^ reported elevated level of SOD activity in *Gambusia affinis* after exposure to monocrotophos. Nwani^41^ reported increased level of SOD activity in *C. carpio* after exposure to herbicide atrazine.

**Figure 5:**
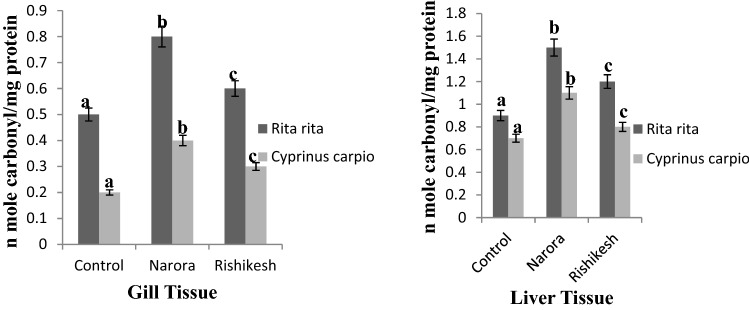
Protein carbonyl content in gill and liver tissues of *Rita rita* and *Cyprinus carpio* from Narora and Rishikesh. The values are expressed as mean ± SE (n = 10). Values are expressed as nanomoles of carbonyl/mg of protein. The significance levels observed is P ≤ 0.05 when compared with the control group.

The pesticides are known to have adverse effects on both environment and its different constituents. Protein content in the organism is main target for these pesticides. Oxidative modification by alteration in physiology and by the impact of different pathological processes may be primary or secondary. Primary modifications are mainly radiation-mediated oxidation, metal catalyzed oxidation^42^. Secondary modifications are based on modification of protein by molecule generated by oxidation of other molecules^42^. In the present study a significant difference in the protein carbonyl content has been observed in both gill and liver tissue of the two fishes (Fig. 5). Protein carbonyl content of gill tissue shows significant increase in *R. rita* and *C. carpio* at both stations. Similarly a significant increase has been observed in liver tissue of the two fish at both the two stations than control group. Among the two stations protein carbonyl content was observed much higher at Narora station. The difference in the protein carbonyl content is mainly due the presence of high pesticide load at the Narora site. Earlier studies by^14,15^ show oxidative stress inducing potential of pesticides in the fish.

The general histological investigations showed low to moderate prevalence of chemical damage on the tissue of the two edible fishes of the river Ganga. Histological changes were recorded in the gill and liver tissues and are summarised in Table 2.

**Table 2:** Summarized histopathological changes in gill and liver tissues of Rita rita and Cyprinus carpio from Narora and Rishikesh site of river Ganga.

Fish	Site	Tissue	Sloughing of Lamellar epithelium and their disruption	Focal area lamellar fusion	Dilated vessel	Lamellar congestion	Gill bridging	Smooth muscle hypertrophy
Rita rita	Narora	Gill	+ +	+	o	+ +	+	o
	Rishikesh		o	+ +	+	+	o	o
Cyprinus carpio	Narora	Gill	+ +	o	+ +	+	o	+
	Rishikesh		+ ++	o	+	o	o	o

Fish	Site	Tissue	Necrotic changes in hepatocytes	Melanomarophage	Focal lymphocytic and macrophage infiltration	Distorted vessel wall	Vacuolation in hepatocytes	Pyknotic nuclei
Rita rita	Narora	Liver	+	+	+	o	o	o
	Rishikesh		o	o	o	o	+++	o
Cyprinus carpio	Narora	Liver	o	o	o	+	++ +	o
	Rishikesh		+	o	o	o	+ +	+

O absent, + low frequency, ++ frequent,  +++ very frequent.

**Figure 6:**
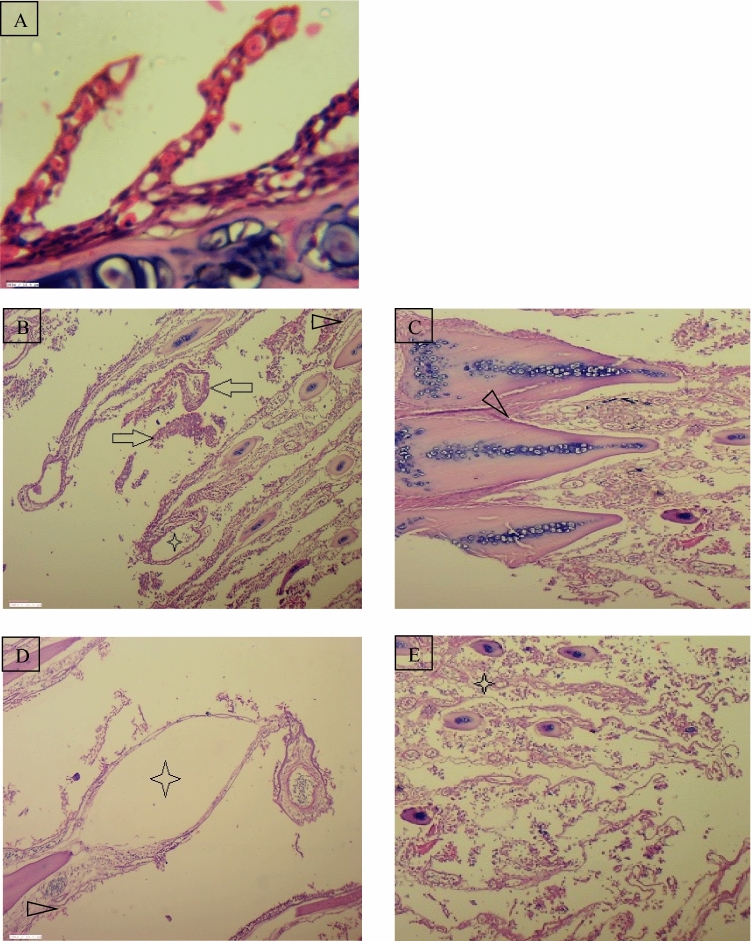
(**A**) Regular shaped secondary gill lamellae in control fish, Gill tissue of *Rita rita* (Narora) (**B**) Sloughing of lamellar epithelium and their disruption (Arrow), Lamellar congestion (Arrow head), (**C**) Gill bridging (Arrow head). Gill tissue of *Rita rita* (Rishikesh), (**D**) Dilated vessel (Star), Lamellar congestion (Arrow head) (**E**) Focal area of lamellar fusion and their disruption (star).

**Figure 7:**
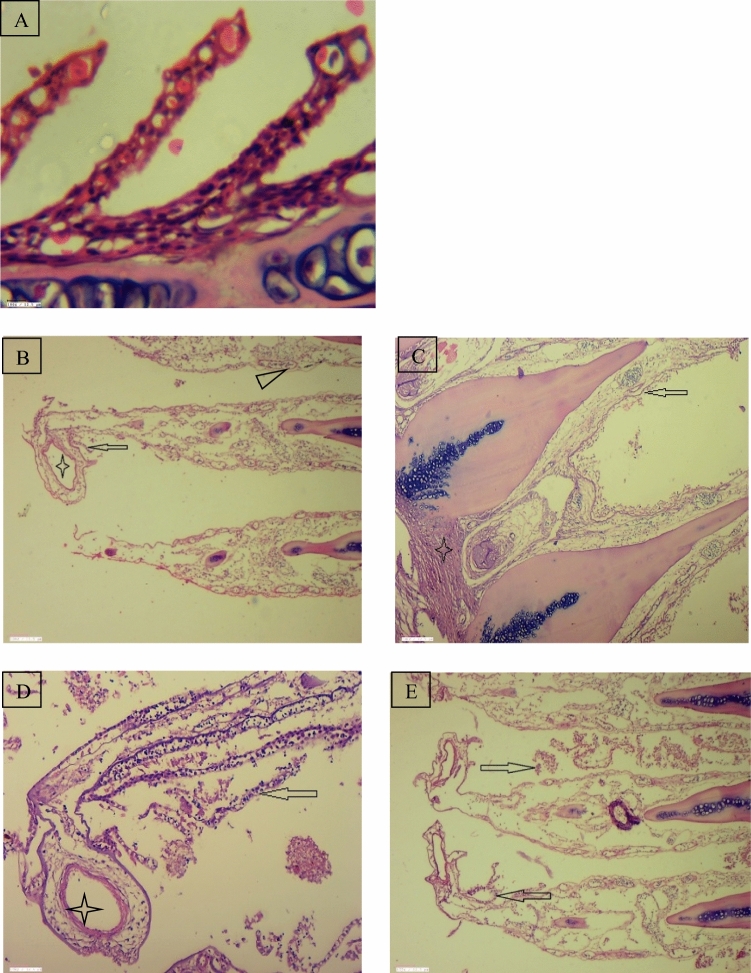
(**A**) Regular shaped secondary gill lamellae in control fish, Gill tissue of *Cyprinus carpio* (Narora) (**B**) Dilated vessel (star), Sloughing of lamellar epithelium and their disruption (Arrow), Lamellar congestion (Arrow head). (**C**) Disrupted lamellar epithelium (Arrow) and smooth muscle hypertrophy (Star). (**D**, **E**) Gill tissue of *Cyprinus carpio* (Rishikesh), Sloughing of lamellar epithelium (Arrow), Dilated vessel (star), Sloughing of lamellar epithelium and their disruption (Arrow).

The hepatocytes and liver cells in the control group were seen normal and systematically arranged with centrally located nucleus. The liver tissues of the fish *R. rita* collected from Narora site revealed different degree of necrotic changes in hepatocytes, melanomarophage, distorted arterial wall and inflammatory cell infiltrate are seen in Fig. 6. The liver tissue of the fish *R. rita* from Rishikesh site of river Ganga shows vacuolation in hepatocytes throughout the liver Fig. 7.

Hepatocytes in the liver are the most prevalent cell type and perform the majority of its key tasks, like conversion of glucose to glycogen, lipid control, and amino acid deamination^43^. Hepatocyte structural damage in reaction to xenobiotics such as pesticides can result in liver dysfunction^44^. The exposed fish's liver had slightly vacuolated hepatocytes with evidence of fatty degeneration necrosis of some liver probably resulted from the excessive work required by the fish to get rid of the toxicant from its body during the process of detoxification by the liver.

**Figure 8:**
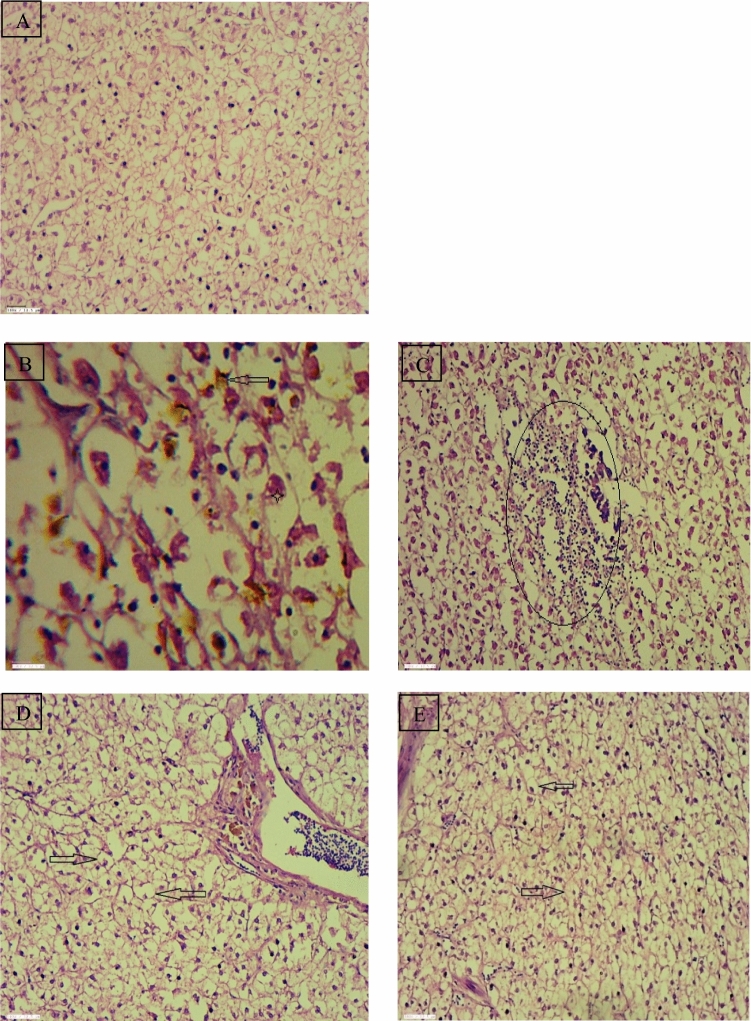
(**A**) Regular round shaped nuclei of hepatocytes of control fish, Liver tissue of *Rita rita* (Narora) (**B**, **C**), Necrotic changes in hepatocytes (Star) and Melanomacrophages (Arrow), Necrotic changes in hepatocytes, focal lymphocytic and macrophage infiltration (Oval). (**D**, **E**) Liver tissue of *Rita rita* (Rishikesh), Vacuolation in hepatocytes (Arrow).

**Figure 9:**
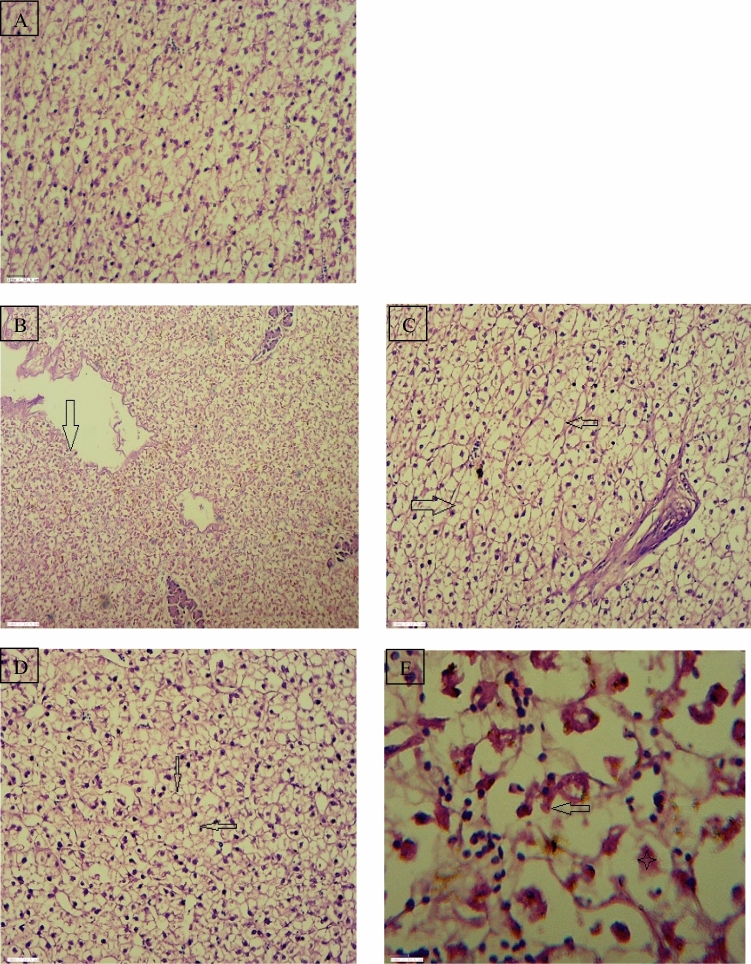
(**A**) Regular round shaped nuclei of hepatocytes of control fish, Liver tissue of *Cyprinus carpio* (Narora) (**B**, **C**), Distorted vessel wall (Arrow), Vacuolation in hepatocytes (Arrow). (**D**, **E**) Liver tissue of *Cyprinus carpio* (Rishikesh), Vacuolation in hepatocytes (Arrow), Necrotic changes in hepatocytes (Star) and Pyknotic nuclei (Arrow).

The control fishes show no histopathological alterations in the gill tissue. Gill filaments and lamellae in both the fishes were seen well structured with flat epithelial cells and pillar cells lining the blood sinusoids. Figure 8 shows the gill of *R. rita* from Narora site displaying varied degree of smooth muscle hypertrophy, sloughing of lamellar epithelium and their disruption, lamellar fusion and their disruption and dilation of vessel. The gill tissue of the *R. rita* at Rishikesh site (Fig. 9) shows dilated vessel, Lamellar congestion, Lamellar swollen and Gill bridging. In the gill tissue of *C. carpio* collected from Narora site shows dilated vessel, sloughing of lamellar epithelium and their disruption, disrupted lamellar epithelium and smooth muscle hypertrophy. The gill tissue of *C. carpio* collected from Rishikesh site shows sloughing of lamellar epithelium, focal area of lamellar fusion and their disruption.

Since gills function as respiratory and osmoregulatory organ of fish, any histopathological change due to toxicity to the gills may impair respiratory function by reducing total respiratory surface area, resulting in hypoxia and respiratory failure problems^45,46^ which has a negative impact on the fish's physiology and may result in death^46,47^. As a result, every change in water quality has a negative impact on histology and functioning of gill.

## Conclusion

The present study showed that, pesticides cause oxidative stress, as evidenced by significant increase in enzymatic antioxidants in the fishes captured from both Narora and Rishikesh station of river Ganga. In *R. rita* and *C. carpio*, changes in protein carbonyl concentration and the use of non-specific histopathological alterations indicate river Ganga is highly polluted at Narora station, thus suggesting them as sensitive and effective tool for reflecting unfavorable environmental conditions for fish health. However, more research is needed to confirm the presence of pesticides and other environmental toxins harming the species in the river under investigation. On the other hand, frequent monitoring and accessing the effect of environmental pollution on total flora and fauna of the river Ganga is important.

## Data Availability

The data that support the findings of this study are available on request from the corresponding author.
